# Summer temperature can predict the distribution of wild yeast populations

**DOI:** 10.1002/ece3.1919

**Published:** 2016-01-27

**Authors:** Heather A. Robinson, Ana Pinharanda, Douda Bensasson

**Affiliations:** ^1^ Faculty of Life SciencesUniversity of ManchesterManchesterM13 9PTUK; ^2^ Department of ZoologyUniversity of CambridgeCambridgeUK

**Keywords:** *Candida albicans*, climate envelope modeling, *Lachancea thermotolerans*, microbial ecology, *Saccharomyces kudriavzevii*, species range, *Wickerhamomyces anomalus*

## Abstract

The wine yeast, *Saccharomyces cerevisiae*, is the best understood microbial eukaryote at the molecular and cellular level, yet its natural geographic distribution is unknown. Here we report the results of a field survey for *S. cerevisiae*,*S. paradoxus* and other budding yeast on oak trees in Europe. We show that yeast species differ in their geographic distributions, and investigated which ecological variables can predict the isolation rate of *S. paradoxus*, the most abundant species. We find a positive association between trunk girth and *S. paradoxus* abundance suggesting that older trees harbor more yeast. *S. paradoxus* isolation frequency is also associated with summer temperature, showing highest isolation rates at intermediate temperatures. Using our statistical model, we estimated a range of summer temperatures at which we expect high *S. paradoxus* isolation rates, and show that the geographic distribution predicted by this optimum temperature range is consistent with the worldwide distribution of sites where *S. paradoxus* has been isolated. Using laboratory estimates of optimal growth temperatures for *S. cerevisiae* relative to *S. paradoxus*, we also estimated an optimum range of summer temperatures for *S. cerevisiae*. The geographic distribution of these optimum temperatures is consistent with the locations where wild *S. cerevisiae* have been reported, and can explain why only human‐associated *S. cerevisiae* strains are isolated at northernmost latitudes. Our results provide a starting point for targeted isolation of *S. cerevisiae* from natural habitats, which could lead to a better understanding of climate associations and natural history in this important model microbe.

## Introduction

The wine yeast, *Saccharomyces cerevisiae*, is of considerable importance to humans for agriculture, industry, and basic research, but little is known about its ecology (Goddard and Greig 2015; Liti 2015). Wild populations of *S. cerevisiae* have been isolated from oak and other tree species in North America, Europe, and Asia (Sniegowski et al. 2002; Sampaio and Gonçalvez 2008; Diezmann and Dietrich 2009; Wang et al. 2012; Hyma and Fay 2013), and are genetically distinct from those associated with human activity (Fay and Benavides 2005; Cromie et al. 2013; Almeida et al. 2015). These woodland habitats and the populations they contain therefore represent a good target for revealing the ecology of *S. cerevisiae*, and the full extent of phenotypic and genetic diversity within the species. A fundamental challenge, however, is that the natural geographic distribution of *S. cerevisiae* is unknown. Indeed, geographic distributions are described for only few individual, free‐living microbial species (Green and Bohannan 2006; Martiny et al. 2006; Taylor et al. 2006). In Portugal and parts of the United States, *S. cerevisiae* is sympatric with *S. paradoxus* (Sniegowski et al. 2002; Sampaio and Gonçalvez 2008; Hyma and Fay 2013). In northern Europe and Canada however, intensive sampling has yielded only *S. paradoxus* (Johnson et al. 2004; Charron et al. 2014; Kowallik et al. 2015; Leducq et al. 2015; Sylvester et al. 2015). Without knowing the expected geographic distribution of the species, wild populations of *S. cerevisiae* remain challenging to find, hindering studies on its natural ecology and genetic diversity.

Experiments in the laboratory show that *S. cerevisiae* has a higher optimum growth temperature than *S. paradoxus* (Sweeney et al. 2004; Salvadó et al. 2011; Leducq et al. 2014). Some aspect of seasonal temperature may therefore predict the differences in the geographic range of these species (Charron et al. 2014; Leducq et al. 2014). It seems unlikely that winter temperatures would be the best predictor of the differences in geographic distributions between the two species as they grow at similar rates at low temperatures (5–23°C; Sweeney et al. 2004; Salvadó et al. 2011). Furthermore, both *S. paradoxus* and *S. cerevisiae* strains isolated from North American oak trees show high tolerance to freezing and thawing (Will et al. 2010). In contrast, *S. cerevisiae* strains grow much faster than *S. paradoxus* at temperatures over 30°C, and *S. cerevisiae* strains are typically able to grow at temperatures over 40°C whereas most *S. paradoxus* cannot (Liti et al. 2009; Salvadó et al. 2011). The optimum growth temperatures for both species (Sweeney et al. 2004; Salvadó et al. 2011) are also similar to maximum summer temperatures in Europe and North America (Hijmans et al. 2005). Therefore, in this study we investigated summer temperature as a potential predictor of the geographic distributions of *S. cerevisiae* and *S. paradoxus*.

We surveyed for the presence of *S. cerevisiae*,* S. paradoxus*, and other budding yeast on oak trees in northern and southern Europe, where summer temperatures are especially low and high. In addition to summer temperature, we considered other ecological variables that might be important in this habitat. For example, ancient oaks seem likely to harbor a much greater diversity of microbes than young trees, and thus, we also collected trunk girth data as a proxy for tree age. We isolated wild *S. cerevisiae* only in southern Europe, and at a rate that was too low for a direct analysis of its distribution. Focusing instead on the distribution of its sister species, *S. paradoxus*, we detected associations between isolation rate, trunk girth, and summer temperature, and used our model of these relationships to estimate the range of summer temperatures where *S. paradoxus* is predicted to be most abundant on oak trees. Using our estimated optimal temperature range for *S. paradoxus* and a laboratory estimate of the difference in temperature preference for woodland *S. cerevisiae* and *S. paradoxus* strains (Sweeney et al. 2004), we predicted the worldwide geographic distributions of optimal summer temperatures for both species. In order to test our predictions, we compiled a dataset of sampling locations and genotype information that includes hundreds of *S. cerevisiae* as well as *S. paradoxus* isolates from previous studies (Naumov et al. 1997; Kuehne et al. 2007; Liti et al. 2009; Zhang et al. 2010; Wang et al. 2012; Cromie et al. 2013; Leducq et al. 2014; Almeida et al. 2015, and references therein). We show that the geographic distribution of *S. paradoxus* and wild *S. cerevisiae* is consistent with the potential ranges that we predict based on their optimal temperatures. We discuss the implications of our results for future field sampling and research into the ecology and evolutionary genetics of these and other yeast species.

## Materials and Methods

### Isolation of yeasts from fruit and oaks

**Figure 1:**
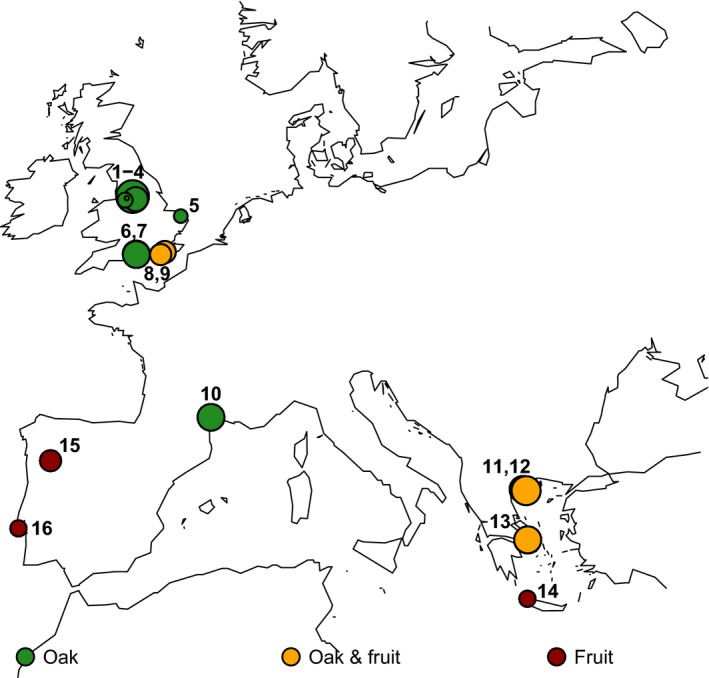
Sample collection sites for yeast strains isolated in this study. Circles are scaled by the natural log of the sample size. Numbers correspond to sites with oak trees in Table 2. No oak trees were sampled at field sites 14–16, and thus, these sites were not included in Table 2.

Between September 2006 and November 2011, we collected 812 environmental samples from oak trees (UK, France, and Greece), fruiting fig trees (Portugal and Greece), vineyard grapes (UK), and garden grapes (Greece; Fig. 1, Tables 1 and 2). The substrates tested for oak were mostly bark (*n* = 618), but a small number of soil samples (*n* = 15) were also collected at the base of some oak trees. The substrates tested for fig and grape were mostly fruit (*n* = 84 and *n* = 53, respectively), but also include fig bark (*n* = 9), grape bark (*n* = 21), and grape must (*n* = 12).

**Table 1:** Yeast species isolated from oaks and fruits in northern and southern Europe

Region^a^	Host	Samples	Sites	Strains	Species
North	Oak	372^b^	9	39	*Saccharomyces paradoxus*
				16	*Lachancea thermotolerans*
				11	*Wickerhamomyces anomalus*
				3	*Candida albicans*
				2	*Hanseniaspora osmophila*
				2	*Hyphopichia burtonii*
				2	*Saccharomycetaceae* sp.
				2	*Saccharomycodes ludwigii*
				1	7 Different *Saccharomycetales* species
South	Oak	261	4	46	*Lachancea thermotolerans*
				44	*Saccharomyces paradoxus*
				4	*Pichia manshurica*
				3	*Saccharomyces cerevisiae*
				2	*Kluyveromyces lactis*
				2	*Meyerozyma sp*.
				1	3 Different *Saccharomycetales* species
North	Grape	57^c^	2	19	*Saccharomyces cerevisiae*
				8	*Wickerhamomyces anomalus*
				2	*Dekkera bruxellensis*
				2	*Saccharomyces paradoxus*
				1	4 Different *Saccharomycetales* species
South	Grape	29	2	4	*Starmerella bacillaris*
				1	4 Different *Saccharomycetales* species
South	Fig	93^d^	4	8	*Meyerozyma* sp.
				6	*Saccharomyces cerevisiae*
				5	*Zygosaccharomyces bailii*
				4	*Saccharomyces* sp.
				3	*Pichia kudriavzevii*
				3	*Starmerella bacillaris*
				1	4 Different *Saccharomycetales* species

a Nine UK sites are classed as northern and seven sites in France, Greece, and Portugal are classed as southern (Fig. 1). Data S2 contains detailed information for all yeast isolates.

b Includes data for 15 soil samples collected at the base of oak trees.

c Includes data for 21 samples from grape vine bark and 12 samples from fermenting grape must.

d Includes data for nine samples from fig tree bark.

**Table 2:** Isolation frequencies of *S. cerevisiae* and *S. paradoxus* from oak bark

Country	Site	Location	Trees^a^	Samples	Mean$T_{max}^{2}$ b	Mean girth^c^	*Sc*	*Sp*	*Sp* freq.^d^
UK	1	Brockholes Wood	15	131	19.6	1.5	0	10	0.08
	2	Chorlton	1	1	21.3	1.1	0	0	0.00
	3	Ladybower Wood	4	32	19.6	2.3	0	7	0.22
	4	Tatton Park	2	5	20.1	4.0	0	1	0.20
	5	Earlham Park	2	3	20.9	6.8	0	1	0.33
	6	Fritham, New Forest	15	60	21.3	3.3	0	7	0.12
	7	Ocknell, New Forest	15	59	21.4	1.5	0	4	0.07
	8	Davenport Vineyard	6	28	21.4	1.3	1	1	0.04
	9	Plumpton Vineyard	6	24	21.6	1.3	0	3	0.12
France	10	Montbarri, Bédarieux	15	59	28.0	0.8	1	9	0.15
Greece	11	Taxiarchis	15	60	27.3	0.8	0	20	0.33
	12	Pyrgadikia	15	82	30.9	1.4	2	14	0.17
	13	Parnitha	15	60	29.7	1.1	0	1	0.02

a Includes data for 22 trees that were excluded from generalized linear models because of missing data for tree trunk girth (see Materials and Methods).

b Average of the daily maximum temperature in the hottest month of the year (°C). Weighted means are shown in cases where $T_{\max}$ of trees differ within a site.

c Weighted mean trunk girth (m), weighted by the number of bark samples per tree.

d For each site, the number of *S. paradoxus* isolates/number of samples.

Host plants were photographed, and longitude and latitude were recorded in WGS84 format (https://github.com/bensassonlab/yeastecology/). Oak trees were classified as *Quercus robur*,* Q. petraea*,* Q. pubescens*,* Q. virgiliana*,* Q. frainetto*, and *Q. ilex* using field guides (Sutton 1990; Fitter and More 2002). As an indicator of oak tree age, we measured trunk girth approximately 1 m above the base of the tree. A number of the oak trees sampled were coppiced, and in these cases, oak girth measurements taken from a single trunk underestimate the age of trees relative to uncoppiced trees. Using photographs of each tree, we treated trunk girth as missing data for 20 trees that were either coppiced or for which we could not determine coppicing status. No girth measurements were taken for an additional two trees sampled. In total, trunk girth data were missing for 22 trees of 126 in our final statistical model.

Using sterile technique, environmental samples were collected from each host plant, stored in tubes for up to a week at room temperature, and weighed upon return to the laboratory. All samples were then incubated for at least two weeks in a liquid medium containing chloramphenicol and 7.6% ethanol that enriches for *Saccharomyces* (Sniegowski et al. 2002). Most samples were incubated at 30°C, but 16 pilot samples were incubated at 10°C, and 18 at 25°C. Aliquots from 7.6% ethanol enrichment medium were streaked onto selective plates with a sole carbon source of methyl‐*α*‐D‐glucopyranoside (Sniegowski et al. 2002), and if weak yeastlike growth was seen on selective plates, then we also streaked from the 7.6% ethanol enrichment medium onto yeast extract peptone glucose (YPD) agar plates.

For each of the yeast‐containing environmental samples, we picked multiple colonies from selective or YPD plates, pooled them in a single YPD liquid culture, and grew these pooled cultures to stationary phase. An aliquot of the pooled colony YPD liquid culture was preserved in 15% glycerol at −80°C, while the rest was used for DNA extraction. This pooled DNA was tested for the presence of our target species, *S. cerevisiae* and *S. paradoxus*, with species‐specific PCR primers. In parallel, for every environmental sample that had yeastlike colonies on the original plates, we also picked a single colony into YPD liquid medium, preserved an aliquot of this single‐colony YPD culture, and identified the yeast species present. If tests on pooled DNA showed that an environmental sample contained *S. cerevisiae* or *S. paradoxus*, but the single‐colony culture contained a different species, then we plated the pooled culture and tested more individual colonies from this or from the original plate until we isolated *S. cerevisiae* or *S. paradoxus*. By testing both pooled samples and single‐colony cultures, it was possible to detect *S. cerevisiae* or *S. paradoxus* when other species were also present, as well as to detect *S. cerevisiae* and *S. paradoxus* in the same samples. As a result, we occasionally isolated *S. cerevisiae* or *S. paradoxus* with other yeast species from single environmental samples (8 of 812 samples).

### Identification of yeast species

DNA was extracted from yeast using the Promega Wizard^®^ Genomic DNA purification kit, according to the manufacturer's instructions for yeast, except that only 75 units of lyticase (Sigma) were typically used in an overnight incubation at 37°C. Conditions for PCR and DNA sequencing were as described in Bensasson (2011). DNA sequencing reads from PCR products were assembled using the Gap4 shotgun assembly tool of Pregap4 version 1.6‐r (Bonfield et al. 1995). Base accuracies were estimated by Pregap4 using its logarithmic (phred) scale. Consensus sequences were all exported from Gap4 (version 4.11.2‐r.) in fasta format. Low‐quality consensus base calls were defined as those with a phred‐scaled quality below q40, and were masked in the consensus sequence as “N." Most DNA sequences (*n* = 300) had more than 200 high‐quality bases and fewer than 100 low‐quality bases and were submitted to NCBI [KT206983–KT207282]. A further 71 DNA sequences did not meet GenBank submission criteria, because they were technical replicates, were less than 200 bases long or contained more than 100 Ns, but were of sufficient quality for species identification and are available at https://github.com/bensassonlab/yeastecology/.

We used rapidly evolving centromeres (CEN6, CEN9, and CEN15) to identify *S. cerevisiae* and *S. paradoxus* strains (Bensasson et al. 2008), and rDNA (18SrRNA‐ITS1‐5.8SrRNA‐ITS2‐25SrRNA) to identify other yeast species. All DNA samples were tested with primers specific to *Saccharomyces* CEN6, one *S. cerevisiae*‐specific primer pair and one *S. paradoxus*‐specific centromere primer pair (CEN6, CEN9, and CEN15; Bensasson 2011; Table S1). In cases where PCR products were amplified using species‐specific CEN primers, we sequenced at least one species‐specific PCR product. All other DNA samples were tested using generic rDNA PCR primers (Table S1), and at least one rDNA sequence was generated for every isolate. We designed generic rDNA primers using primer3 (http://primer3.sourceforge.net/) that would anneal to all known Saccharomycetales rDNA sequences (in NCBI, June 2007), including 15 different Debaryomycetaceae and Saccharomycetaceae species.

Each isolate was then classified on the basis of the similarity of its centromere or rDNA to known yeast species using NCBI BLAST (https://blast.ncbi.nlm.nih.gov/). Every DNA sequence was queried against the nucleotide collection (nr/nt, date: August 28th, 2015) database restricted to the Ascomycota (taxid: 4890), excluding a strain with *Lachancea thermotolerans* rDNA sequence that was classified as *S. paradoxus* in GenBank (Entrez Query “NOT LL12_027"). Searches were performed using the blastn algorithm (version 2.2.32+), with an expect threshold of 0.001, and no filtering for low‐complexity regions. BLAST output was parsed using a custom Perl script to extract the species names for hits with the highest BLAST score, and to assign species given a set of species name synonyms defined in the NCBI taxonomy (Data S2. For most yeast isolates (*n* = 247), species assignment was unambiguous; all hits with the highest BLAST score belong to only a single species (sometimes with multiple synonyms), and we assumed this was the species isolated. For a few strains (*n* = 17), DNA sequence had equal BLAST scores for multiple species, and in these cases, we could only assign species to genus or higher taxonomic levels.

### Statistical analysis

All statistical and graphical analyses were conducted in R, version 3.1.1. Maps were drawn using the raster (version 2.3‐40) and maps (version 2.3‐9) packages using summer temperature ($T_{\max}$) data from the WorldClim dataset version 1.4 (1950–2000, release 3, http://www.worldclim.org) at 10 arc‐minute (Fig. 4) or 30 arc‐second (approximately 1 km) resolution (Fig. 5, Figure S1, Data S3 and S4; Hijmans et al. 2005). $T_{\max}$ was estimated using raster for every host plant from a single pixel at 30 arc‐second resolution. $T_{\max}$ in the WorldClim dataset is the daily maximum temperature, averaged over the hottest month of the year (Robert Hijmans, personal communication).

Using a generalized linear model (GLM) with binomial errors, we modeled *S. paradoxus* isolation frequency by setting the proportion of bark samples with *S. paradoxus* from an oak tree as the response variable. The initial model included four explanatory variables and all their possible interactions: (i) trunk girth (in meters) as a continuous variable; (ii) $T_{\max}$ (in °C × 10) as a continuous variable estimated from a single pixel at 30 arc‐second resolution given the longitude and latitude of each tree; (iii) a three‐level factor describing oak type as robur‐like (the northern *Q. robur* or *Q. petraea*), frainetto‐like (the southern *Q. frainetto*,* Q. pubescens* or the intermediate *Q. virgiliana*) or the outgroup species *Quercus ilex*; and (iv) a continuous variable describing the frequency of non‐*S. paradoxus* yeast species isolation (the number of other yeast species isolated divided by the number of samples collected for each tree). This initial model was simplified by subtracting terms in a stepwise manner starting from the highest order terms and testing whether each subtraction resulted in a worse model using chi‐square tests as recommended in Crawley (2005). The three‐level factor for oak type was then further simplified to two levels and nested models were again compared using chi‐square tests following the principles for model simplification by contrasts described in Crawley (2005).

**Figure 2:**
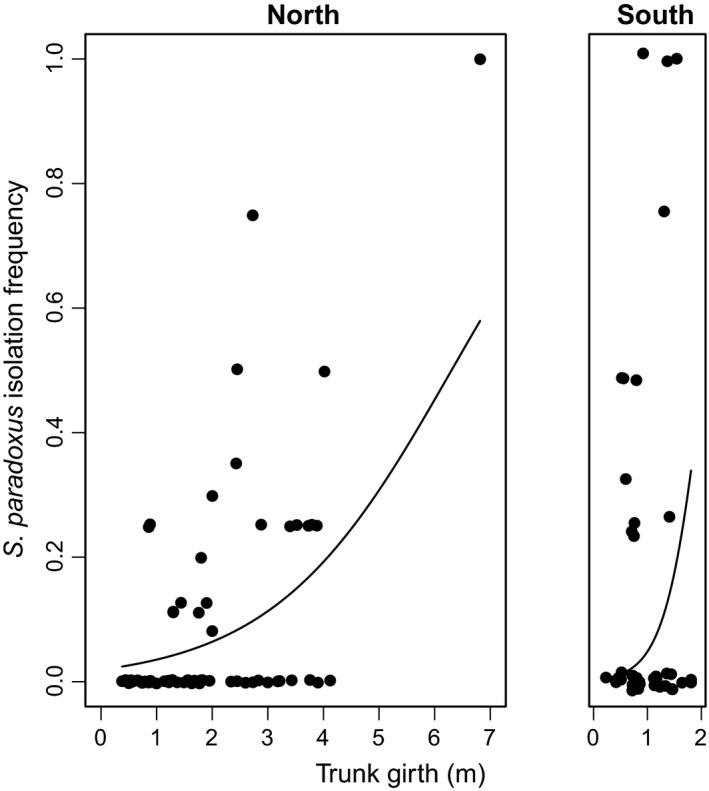
*S. paradoxus* isolation frequency increases with trunk girth. Points show the observed isolation frequencies for 104 trees from northern (UK) and southern Europe (France and Greece). For each tree, we estimated the frequency of *S. paradoxus* isolation as the number of pieces of bark yielding *S. paradoxus* divided by the number of pieces of bark sampled. Points are clustered around discrete frequencies because in most cases the number of pieces of bark sampled was four. We therefore used jitter to allow better visualization of data. Lines show the probability of isolating *S. paradoxus* estimated from the final GLM assuming median summer temperatures in northern ($T_{\max}$ = 21.3°C) and southern Europe ($T_{\max}$ = 28.6°C).

**Figure 3:**
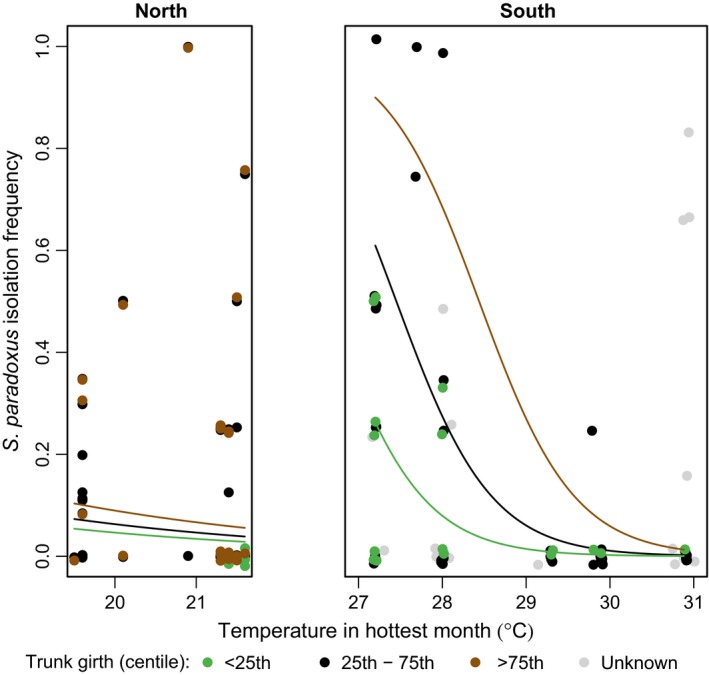
The effects of temperature in the hottest month on *S. paradoxus* isolation frequency. *S. paradoxus* isolation frequency is estimated as the proportion of bark samples from each tree with *S. paradoxus*; more specifically, the number of *S. paradoxus* isolates for a tree divided by the total number of bark samples obtained for that tree. Points show the distribution of the data, including points for which no trunk girth data are available (gray, see Materials and Methods). Jitter was used to better display overlapping points. Lines show the predicted probability of isolating *S. paradoxus* and are estimated from the final generalized linear model given lower (0.8 m), median (1.3 m), and upper (1.9 m) quartile measurements of tree trunk girth (green, black, and brown, respectively).

Both the initial and final models showed expected levels of deviance given the number of degrees of freedom (final model, residual deviance = 75, df = 98). Cook's distance analysis was also used to identify the trees with the highest influence on the parameter estimates of the model. As a control we investigated the effects of each of these data points on the analysis, and found the removal of single data points did not qualitatively change the final model. To control for the possibility that a single site in southern Europe affects our conclusions, we investigated the effects on the analysis of dropping all data for one southern field site at a time. In all cases, we observed all the same statistically significant effects (*P* < 0.04), and visualization of the effects showed no qualitative difference from the results shown in Figures 2 and 3.

### Worldwide presence and absence data for *S. paradoxus* and *S. cerevisiae*

In order to test whether *S. cerevisiae* and *S. paradoxus* have been isolated from locations with summer temperatures within the optimum ranges that we predict, we needed sample location and genotype information for a large number of strains. Sampling locations have been mapped for thousands of yeast strains from many species that have been deposited in the Centraalbureau voor Schimmelcultures collection (Robert et al. 2006; Kurtzman et al. 2015). This resource is not available for download however, and does not provide genotype information, which we need in order to distinguish wild from human‐associated *S. cerevisiae* strains. Location information has been mapped together with genotype information for *S. paradoxus* (Boynton and Greig 2014), but not for *S. cerevisiae*.

Therefore, we collated site location information together with genotype information from previous studies on *S. cerevisiae* (Zhang et al. 2010; Wang et al. 2012; Cromie et al. 2013; Almeida et al. 2015) and *S. paradoxus* (Naumov et al. 1997; Kuehne et al. 2007; Liti et al. 2009; Zhang et al. 2010; Leducq et al. 2014). No data for *S. paradoxus* strains isolated in this study that were used in the construction of our statistical model were included in this validation dataset. Site location and genotype information for *S. cerevisiae* strains isolated as part of this study were included, because no information for these strains was used to generate the model. The criteria for including data from a study were that it provided genotype information for many strains (that are not already included in a larger study) and it included strains isolated from substrates that are not wine or vineyard grapes. In most previous studies, latitude and longitude information was not included in site descriptions. We therefore used site descriptions as search terms in Google Maps. Where site descriptions map to a large region, we used latitude and longitude coordinates from the estimated center of that region. Data for yeast strains with site descriptions that did not allow location within 100–200 km were excluded (e.g., strains from unknown locations or with their origin described as “Europe"). We also excluded strains isolated from wine or vineyard grapes, because we expect that their distribution is affected by human activity (Fay and Benavides 2005). *S. cerevisiae* was also recorded as absent from several sites where surveys of over 100 bark samples yielded no *S. cerevisiae*: site 1 from this study (Table 2), Johnson et al. (2004), Charron et al. (2014) and Kowallik et al. (2015).

$T_{\max}$ was estimated for every isolate using the raster package from a single pixel at 30 arc‐second resolution. For collection sites that occur at locations with summer temperatures outside the range that we predict with our statistical model, we estimated the distance to regions that are within the expected range. The regions in which such sites occurred were visualized using the raster and maps packages in R, and the distance (in kilometers) was estimated using the sp package in R (version 1.1‐1).

## Results

### Variation in the geographic distribution of yeast species

We conducted a field survey with the aim of isolating yeast species from the *Saccharomyces sensu stricto* genus, and isolated 264 yeast strains from 812 European oak, fig, and grape samples (Table 1, Fig. 1, Data S3). These strains are from at least 26 different yeast species across the order Saccharomycetales, including 5 different yeast families: Saccharomycetaceae, Saccharomycodaceae, Debaryomycetaceae, Phaffomycetaceae, and Pichiaceae (Data S2). Although it is rarely isolated in natural environments (Tanghe et al. 2005; Lachance et al. 2011; Maganti et al. 2011), we isolated three strains of the human commensal and pathogen, *Candida albicans* from ancient oak trees in northern Europe (site 6 in Fig. 1 and Table 2, Data S1). *C. albicans* has only rarely been isolated away from mammals (Tanghe et al. 2005; Lachance et al. 2011; Maganti et al. 2011), and the existence of wild populations of *C. albicans* on north European trees could potentially explain the hitherto puzzling maintenance of aquaporin genes that confer freeze tolerance in *C. albicans* (Tanghe et al. 2005).

The most commonly isolated *Saccharomyces* species was *S. paradoxus*, which we isolated mostly from oak bark and from soil at the base of oak trees (83 of 633 samples, Table 1). We isolated *S. cerevisiae* strains from 25 of 179 fruit, fruit tree bark and grape must samples, but relatively few from oak‐associated samples (4 of 633, Table 1). In addition, we isolated a single strain of *S. kudriavzevii* from oak bark in Greece (site 12, Fig. 1) as well as four strains of a *Saccharomyces sensu stricto* species from figs at the same site that we could not identify to the species level using our methods (Table 1). The greater prevalence of *S. cerevisiae* on fruit trees relative to oaks could however be an effect of geography and human influence, because fruit trees were only sampled in the far south of Europe or in vineyards (Fig. 1, Table 2). Indeed, when we controlled for the effects of geography by considering only sites where *S. cerevisiae* was present, we saw very similar isolation rates from fruit, fruit tree bark and oak bark (Data S1). Others have also observed similar or lower isolation rates from fruit relative to woodland substrates (Wang et al. 2012), and this finding lends support to the proposal that *S. cerevisiae* is not more adapted to fruit than other habitats (Goddard and Greig 2015).

In the UK, we isolated 39 *S. paradoxus* from 372 oak bark and soil samples (Table 1). This isolation rate (10%) is similar to that previously reported for *S. paradoxus* both in the UK (Johnson et al. 2004; 28 isolates from 344 oak bark samples, Fisher's exact test, *P* = 0.3) and Pennsylvania, USA (Sniegowski et al. 2002; 8 of 79 oak bark and soil samples, Fisher's exact test, *P* = 1). In contrast, we isolated fewer *S. cerevisiae* from oak samples in the UK (1/372) than Sniegowski et al. (2002) did from oak trees in Pennsylvania (10/79; Fisher's exact test, $P\;=\;2\;\times \;10^{-7}$), even though we used the same enrichment culturing method and sampled in the same season. The fact that we were able to reproduce the *S. paradoxus* isolation rate, but not the *S. cerevisiae* isolation rate (Sniegowski et al. 2002), suggests a geographic difference in the distribution of *S. cerevisiae* relative to *S. paradoxus*, with a lower abundance of *S. cerevisiae* in the UK than in Pennsylvania.

Analysis of all 264 strains isolated from all 812 European samples suggests that there are also differences in the geographic distributions of other yeast species within Europe (Table 1). In general, we were able to isolate and identify more yeast strains from southern than from northern European oak bark (104/261 compared to 84/372, Fisher's exact test, $P\;=\;4\;\times \;10^{-6}$). This effect is especially strong for *Lachancea thermotolerans*, a yeast common in oak bark (Sampaio and Gonçalvez 2008; Sylvester et al. 2015), which is more common in southern (46 of 261) than in northern oak bark and soil samples (16/372; Fisher's exact test, $P\;=\;4\;\times \;10^{-8}$, Table 1). Previous studies have shown enrichment culturing at different temperatures (10°C compared to 22–30°C) results in the isolation of different yeast species (Sampaio and Gonçalvez 2008; Sylvester et al. 2015). Therefore the bias toward southern yeast distributions might simply be a consequence of the temperature we use for enrichment culturing (25–30°C). However, it is not a universal rule that all yeast species have higher isolation rates in southern versus northern locations. Notably, *Wickerhamomyces anomalus*, a food spoilage yeast that can also contribute to wine aroma (Passoth et al. 2006), was common in northern oak (11 of 372 bark and soil samples) and fruit, but was absent from southern oak bark samples (0/261; Fisher's exact test, *P* = 0.004) and fruit (Table 1).

### Trunk girth and summer temperature can explain differences among oaks in *S. paradoxus* abundance

The original aim of this study was to model the ecological factors affecting the prevalence of *S. cerevisiae* in woodlands, but consistent with other studies on northern European sites (Johnson et al. 2004; Kowallik et al. 2015), we were unable to isolate many *S. cerevisiae* strains from European oaks. Instead, we focused our modeling efforts on its closest relative *S. paradoxus*, which was the most commonly isolated species in this study (Tables 1 and 2). For these analyses, we used data for 78 strains of *S. paradoxus* isolated from 126 oak trees resulting from a total of 604 oak bark samples (Table 2). An average of 4.8 pieces of bark were collected from each tree, and in most cases (87 trees), we collected exactly 4 pieces per tree. To reduce potential variation resulting from experimental procedures, we excluded pilot data for 14 oak bark samples that were incubated at 10°C during enrichment culturing and 15 soil samples collected at the base of oak trees. Analysis of all 604 oak bark samples (Table 2) showed that isolation rates are not affected by collection month and bark sample weight in this study (Data S1), and therefore these variables were not included in our final model. We collected most samples (75%) between 25th August and 7th September, therefore it is unsurprising that we did not detect the seasonal variation that others have observed for *S. paradoxus* abundance (Glushakova et al. 2007; Charron et al. 2014).

Laboratory studies suggest that *S. cerevisiae* and *S. paradoxus* have different temperature preferences for their optimal growth (Sweeney et al. 2004; Salvadó et al. 2011) and also differ in their tolerance of high temperatures (Liti et al. 2009). Therefore, we asked whether summer temperature ($T_{\max}$) can predict the distribution of *S. paradoxus*, in conjunction with other variables that could affect the prevalence of yeast on oak trees, such as host species or tree age. Because other yeast species could potentially outcompete *S. paradoxus* in culture and affect our estimation of its isolation rate, we also consider the presence of other yeast species isolated from each tree in our analysis. Using trunk girth as a proxy for tree age, and binning tree species into three groups (robur‐like, frainetto‐like, and *Q. ilex*; see Materials and Methods), we constructed a generalized linear model (GLM) to test whether the frequency of *S. paradoxus* isolation from an oak tree can be predicted by four explanatory variables (i) trunk girth, (ii) summer temperature, (iii) host tree type, and (iv) isolation frequency of other yeast species.

After standard model simplification (Crawley 2005), we found that the presence of other yeast species does not affect the number of *S. paradoxus* isolated (GLM, −0.02% deviance, df = 1, *P* = 0.9). This suggests that competition among yeast during our isolation procedure does not substantially affect the rate or pattern of *S. paradoxus* isolation. However, all three other explanatory variables are important for predicting numbers of *S. paradoxus* isolated from oak trees. We also found that a simpler final model where oaks are classed as northern or southern is not worse than the model describing three host types (GLM, −2% deviance, df = 3, *P* = 0.4). This suggests that more general differences between northern and southern European field sites can explain differences in *S. paradoxus* yield better than host tree type.

The final GLM explains 42% of the deviance among trees in *S. paradoxus* isolation frequency in terms of tree trunk girth, summer temperature, and whether a site is northern or southern. Trunk girth is an important predictor of *S. paradoxus* isolation frequency, which if dropped leads to a much worse model fit (GLM, −21% deviance, df = 2, $P\;=\;1\;\times \;10^{-6}$). Indeed, if we remove trunk girth data from the analysis, we find that none of the other significant effects in the model would have been detected, suggesting that host tree age is a crucial factor to consider in order to discover variables that are relevant to yeast ecology. As trunk girth increases, *S. paradoxus* isolation frequency increases in northern and southern Europe (Fig. 2). The positive association between trunk girth and the presence of *S. paradoxus* suggests that old oak trees harbor more *S. paradoxus*.

The best predictor of the *S. paradoxus* isolation frequency for a tree was whether it was from northern or southern Europe. Trees from southern Europe yielded more *S. paradoxus* isolates, even though we sampled more trees and larger trees from northern Europe (Table 2, Fig. 3). This effect is especially clear in Figure 3 from the low isolation frequency of *S. paradoxus* that the model predicts in northern Europe compared to the high frequency expected at temperatures around 27–28°C in southern Europe.

There is also a difference between northern and southern trees in the effect of trunk girth on *S. paradoxus* isolation frequency (GLM, −6% deviance df = 1, *P* = 0.004). More specifically, the numbers of *S. paradoxus* isolated from southern oaks increased more steeply with increasing trunk girth than they did from northern oaks (Fig. 2). While trunk girth may be a good proxy for tree age when comparing trees from the same site, it is probably a much worse predictor when comparing multiple species of oak that are growing in differing climatic conditions. Such differences may explain the larger effect of trunk girth on *S. paradoxus* isolation frequency in the south compared to the north (Fig. 2).

In southern Europe, we also observe a negative relationship between *S. paradoxus* abundance and summer temperature, whereas there is no such effect in the north (GLM, −9% deviance, df = 1, *P* = 0.0006, Fig. 3). This suggests that the hottest field sites in southern Europe ($T_{\max}$, 28–31°C) are hotter than the optimum habitat for *S. paradoxus*, which is consistent with laboratory observations of suboptimal growth for most strains of *S. paradoxus* at temperatures over 30°C (Sweeney et al. 2004; Salvadó et al. 2011; Leducq et al. 2014).

Figure 3 shows the predictions of the final model with all the variables of major effect combined. The low predicted *S. paradoxus* isolation frequency between 18 and 22°C suggests an optimum summer temperature for *S. paradoxus* that is higher than 22°C, whereas the negative association between $T_{\max}$ and isolation rate between 28 and 31°C suggests that the optimum is lower than 28°C. Thus, the optimum summer temperature for *S. paradoxus* appears to be between 22 and 28°C.

### Summer temperature can predict the worldwide distribution of wild *S. paradoxus* and *S. cerevisiae* populations

**Figure 4:**
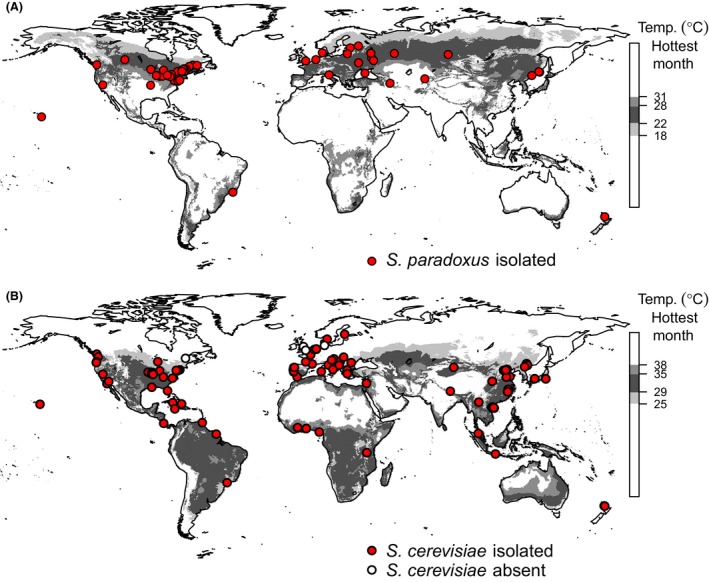
Global distribution of the predicted optimum temperature range for (A) *S. paradoxus* and (B) *S. cerevisiae*. Optimum temperatures for *S. paradoxus* are estimated from Figure 3, and for *S. cerevisiae* we assume the optimum is approximately 7°C higher than that of *S. paradoxus* (Sweeney et al. 2004). Red circles show the approximate origin of strains published in large genotyping studies (Naumov et al. 1997; Kuehne et al. 2007; Liti et al. 2009; Zhang et al. 2010; Wang et al. 2012; Cromie et al. 2013; Leducq et al. 2014; Almeida et al. 2015; and references therein). Location and genotype (Almeida et al. 2015) information from this study is included for *S. cerevisiae* strains but not for *S. paradoxus*, because data for *S. paradoxus* were used to generate our predictions. White circles show locations where surveys of over 100 bark samples yielded no *S. cerevisiae* and are summarized from this study, Johnson et al. (2004), Charron et al. (2014) and Kowallik et al. (2015).

Our analysis of oak bark samples collected from thirteen European sites in the UK, France, and Greece (Table 2, Fig. 3) suggests that the optimum summer temperature ($T_{\max}$) for *S. paradoxus* lies between 22 and 28°C, but that this species is also found at lower abundances between 18 and 31°C (Fig. 3). We tested the predictions of our model by mapping the global distribution of this thermal optimum, and comparing it to sites where *S. paradoxus* has been reported in previous studies (Naumov et al. 1997; Kuehne et al. 2007; Liti et al. 2009; Zhang et al. 2010; Leducq et al. 2014). Virtually all the *S. paradoxus* strains that we mapped from other studies (244 of 246) fall within our predicted range of optimum summer temperatures between 18 and 31°C (Fig. 4A). Indeed, 75% of these *S. paradoxus* strains map to locations where $T_{\max}$ is between 22 and 28°C, and 95% occur between 20 and 30°C. We identified only two strains that could fall outside the $T_{\max}$ range of 18–31°C. One was from Tashkent in Uzbekistan (Naumov et al. 1997), a site that we approximately mapped to the center of Tashkent (with a °C). This approximate mapping is within 30 km of high elevation regions that have a lower summer temperature ($T_{\max}$ of 28°C), which is within our predicted optimum range. The other exception was a strain of *S. paradoxus* isolated from insect excrement (from Missouri, USA, 32°C $T_{\max}$; Leducq et al. 2014), collected over 200 km from locations with temperatures within the predicted range. In addition, *S. paradoxus* strains have been isolated from other parts of Missouri (31.5–32.1°C $T_{\max}$), albeit less often than from Oregon (27°C $T_{\max}$), and at lower frequency than *S. cerevisiae* (Hyma and Fay 2013). Therefore 31°C as an upper limit for *S. paradoxus* isolation (Fig. 3) is probably a slight underestimate.

Ideally, we would like to map the worldwide distribution of the model eukaryote, *S. cerevisiae*. We can make progress toward this goal by combining our results from *S. paradoxus* with the finding by Sweeney et al. (2004) that in the laboratory, *S. cerevisiae* from oak trees grow optimally at roughly 7${}^{\circ }$C higher temperatures than *S. paradoxus*. We use the estimate of the species difference in temperature preferences by Sweeney et al. (2004), because this study uses a large number of *S. cerevisiae* and *S. paradoxus* strains from the same oak habitat, with growth profiles that are typical for their species (see Data S1 for a full discussion). In order to predict the potential geographic range of *S. cerevisiae*, we therefore added 7${}^{\circ }$C to our climate envelope model for *S. paradoxus* to generate a global distribution map based on predicted optimum temperatures for *S. cerevisiae* (Fig. 4B). The potential range that we predict for *S. cerevisiae* is mostly subtropical or tropical and different from the prediction of a temperate distribution for *S. paradoxus* (Fig. 4). Indeed, the predicted worldwide range of *S. cerevisiae* is more consistent with the distribution of *S. cerevisiae* isolates than that of *S. paradoxus*. Specifically, many *S. cerevisiae* strains map to tropical parts of Africa, Southeast Asia, North America, Israel and the Caribbean that are outside the range we predict for *S. paradoxus* (Fig. 4B).

Human culture and transport of *S. cerevisiae* across the world has affected the distribution of this species (Fay and Benavides 2005; Liti et al. 2009; Wang et al. 2012; Cromie et al. 2013). Therefore, when testing the predicted distribution of optimum summer temperature for *S. cerevisiae*, we need to distinguish strains that are associated with human activity from wild strains. Strains associated with human activity, such as those cultured in breweries or vineyards, can potentially escape and survive in regions with otherwise unsuitable climates as feral strains, but these are likely to represent transient (sink) populations. The locations of sink populations do not accurately test the predictions of climate envelope models (Araújo and Peterson 2012). Feral *S. cerevisiae* strains are expected to have genotypes associated with human activity, such as the genotype associated with wine production, or to be “mosaic" strains showing recent genomic admixture between natural populations (Fay and Benavides 2005; Liti et al. 2009; Wang et al. 2012; Cromie et al. 2013; Almeida et al. 2015).

**Figure 5:**
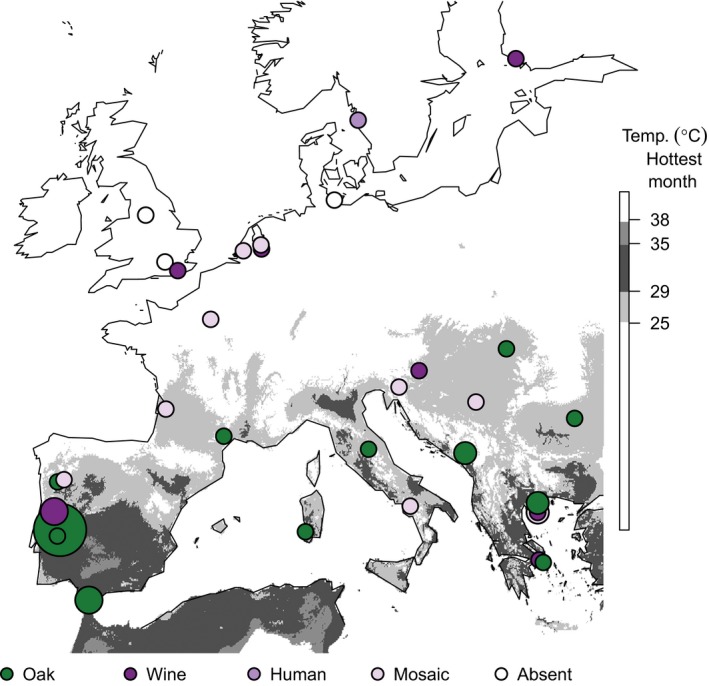
Only feral *S. cerevisiae* or those with mosaic genotypes occur outside the predicted optimal temperature range. The regions with average temperature in the hottest month where we expect *S. cerevisiae* are shaded in gray, assuming it correlates with a 7${}^{\circ }$C higher average temperature in the hottest month than *S. paradoxus* (Sweeney et al. 2004). White points show the locations where over a hundred pieces of bark yielded no *S. cerevisiae* (Johnson et al. 2004; Kowallik et al. 2015; This study). The remaining points show the geographic sources of 46 *S. cerevisiae* strains isolated from various sources that include trees, soil, fruits, and beer (but not including wine or grapes), and are colored by genotype (see Results; data from Cromie et al. 2013; Almeida et al. 2015). Points are scaled by the square root of sample size and two points in Greece were repositioned slightly so that all overlapping points are visible.

The majority of *S. cerevisiae* isolates (222 of 301 strains) from most of the collection sites (71 of 92 sites) that we were able to map worldwide, mapped approximately to locations with summer temperatures within the optimum range that we predict for *S. cerevisiae* (25–38${}^{\circ }$C). Almost half the collection sites outside our predicted range occur in Europe (10 of 21 sites) where yeast sampling intensity is relatively high (Robert et al. 2006; Kurtzman et al. 2015). Figure 5 shows all the *S. cerevisiae* strains (*n* = 46) isolated from Europe with points colored according to genotype. Two distinct genetic lineages of *S. cerevisiae* predominate within Europe (Cromie et al. 2013; Almeida et al. 2015); one is associated with humans and wine and another is associated with oak trees (Almeida et al. 2015) and perhaps also olive trees (Cromie et al. 2013). The vast majority of European *S. cerevisiae* with the wild genotype expected on oak trees (23 of 26 strains) map to locations with summer temperatures within the range that we predict for *S. cerevisiae* (between 25 and 38${}^{\circ }$C, Fig. 5). The three wild strains in Europe that we mapped to locations outside the predicted range of summer temperatures mapped to Mount Subasio in Italy and Jasenovo Polje in Montenegro (Fig. 5). The locations for both of these sites were mapped approximately, and both occur in mountain regions with expected summer temperatures at lower elevation (within 3 km). In contrast, several European strains with human‐associated genotypes (7 of 20 strains) occur at sites that are far from the predicted summer temperatures for *S. cerevisiae* (200–1300 km away). Many of these strains with human‐associated genotypes were isolated from locations that suggest a recent association with humans or that they could represent transient populations: a vineyard tree, buttermilk, a fish's gut, and soil at an agricultural college. It therefore appears that in Europe, *S. cerevisiae* strains that fell outside our predicted range were either rare strains with wild genotypes that were probably incorrectly mapped to higher elevations in mountain ranges, or more commonly human‐associated *S. cerevisiae* that can occur at locations far from our predicted range (Fig. 5).

The patterns that we see in Europe are similar to those we see worldwide. *S. cerevisiae* strains have been isolated from soil, vine bark and buttercups in a New Zealand vineyard (Goddard et al. 2010) outside the predicted range of summer temperatures (24°C, Fig. 4B). These strains have genotypes similar to those of European rather than Asian *S. cerevisiae* (Cromie et al. 2013) and thus may also represent vineyard‐associated sink populations. Of 122 *S. cerevisiae* strains with human‐associated genotypes mapped worldwide, 38 strains occur at locations with summer temperatures that are lower than those we predict for *S. cerevisiae*, and 36 of these are more than 20 km from locations with expected temperatures (Fig. 5, Data S4). In contrast, the 41 of 179 *S. cerevisiae* strains with wild genotypes outside the predicted range were much closer to locations within the predicted range than those with human‐associated genotypes (Wilcoxon test, $P\;=\;9\;\times \;10^{-14}$). All 41 wild *S. cerevisiae* strains that were out of range were mapped only approximately, and 40 of these mapped to mountain locations in Europe and China that were within 8 km of the predicted range (median distance = 1 km; Fig. 5 and Figure S1). The only exception of a strain with a wild genotype occurring far out of range was isolated from a flower in Seattle ($T_{\max}$ 23°C, 84 km from the nearest site within range; Cromie et al. 2013). We therefore conclude that the distribution of wild *S. cerevisiae* strains is consistent with our predicted range.

In addition, our model correctly predicts most of the differences and similarities in the ranges of *S. cerevisiae* and *S. paradoxus*. The difference in the optimum summer temperatures illustrated in Figure 4 can explain the presence of *S. paradoxus* and the absence of *S. cerevisiae* in the UK ($T_{\max}$ 20°C, This study; 23°C; Johnson et al. 2004), Canada ($T_{\max}$ 25°C, Charron et al. 2014) and northern Germany ($T_{\max}$ 21°C, Kowallik et al. 2015). Conversely, the optimum summer temperatures for the two species overlap between 25 and 31°C, where we might therefore expect their sympatry: for example, in the northern United States, parts of southern Europe, northern China, southeastern Brazil, South Africa, and southern Australia. In the northern United States ($T_{\max}$ 30°C; Sniegowski et al. 2002), and southern Europe at least ($T_{\max}$ 31°C, Sampaio and Gonçalvez 2008; Table 2), these prediction are met.

## Discussion

By intensively sampling *S. paradoxus* from oak trees in northern and southern Europe (Fig. 1, Data S3), we discovered associations between *S. paradoxus* isolation frequency, trunk girth (Fig. 2) and summer temperature (Fig. 3). Using the association of *S. paradoxus* with summer temperature in Europe, we predict regions where *S. paradoxus* and *S. cerevisiae* might occur worldwide (Fig. 4). The worldwide distribution predicted by the optimum $T_{\max}$ for *S. paradoxus* is consistent with the observed distribution of *S. paradoxus* isolations from previous studies (Boynton and Greig 2014; Fig. 4A, Data S4), and with the detection of a northern limit to its distribution in Canada (Charron et al. 2014; Leducq et al. 2015). Similarly, our predicted optimum summer temperature for *S. cerevisiae* could potentially explain the success or failure to isolate *S. cerevisiae* in previous studies (Fig. 4B and Data S4; Johnson et al. 2004; Charron et al. 2014; Kowallik et al. 2015), and why *S. cerevisiae* strains isolated outside this range often have human‐associated or mosaic genotypes indicative of transient populations (Fig. 5 and Data S4).

Population genetic analyses show that the genetic diversity of *S. cerevisiae* is exceptionally high in the tropics and subtropics of China (Wang et al. 2012; Almeida et al. 2015), and is unusually low in Europe (Almeida et al. 2015). The genetic diversity of a population is expected to increase as its habitat area increases (Rauch and Bar‐Yam 2005). High genetic diversity of *S. cerevisiae* in China is therefore compatible with the larger potential habitat area we predict in east Asia (Fig. 4B), while low genetic diversity within Europe is consistent with the restricted range predicted for *S. cerevisiae* in Europe (Fig. 5). An alternative explanation for the high genetic diversity of *S. cerevisiae* in China is an east Asian origin for the species (Wang et al. 2012; Almeida et al. 2015). It is currently unknown whether other subtropical or tropical forest populations of *S. cerevisiae* have high genetic diversity as yeasts have been less intensively sampled from such regions (Robert et al. 2006; Kurtzman et al. 2015). Without further sampling in tropical and subtropical regions it is not possible to differentiate whether the higher diversity of *S. cerevisiae* in Asia reflects a greater habitat area or an Asian origin for *S. cerevisiae*.

Although our predictions fit well with the data currently available, this analysis represents only a starting point for understanding the ecological factors controlling the distribution of *S. paradoxus* and *S. cerevisiae*. In this study, we focused only on $T_{\max}$ as a climate variable because laboratory experiments suggest a difference between *S. paradoxus* and *S. cerevisiae* in their growth at high temperatures (Sweeney et al. 2004; Liti et al. 2009; Salvadó et al. 2011; Leducq et al. 2014), but not at low temperatures (Sweeney et al. 2004; Will et al. 2010; Salvadó et al. 2011). Different climate variables are highly correlated within Europe, and using only the field sites in this study (Table 2), we cannot distinguish the association of *S. paradoxus* isolation frequency with summer temperature from associations with other factors such as rainfall or winter temperature. Furthermore, our observation of a negative association between $T_{\max}$ and *S. paradoxus* isolation frequency is based on analysis of data from only four independent field sites in southern Europe. While temperature differences can explain the major differences among our field sites (Data S1), our conclusions would be strengthened by independent verification of the upper limit of the optimum $T_{\max}$ for *S. paradoxus* from additional sites. Thus, while we conclude that summer temperature can predict the range of *S. paradoxus* and *S. cerevisiae*, we do not claim that summer temperature is the causal factor limiting the distribution of *Saccharomyces* species.

In the case of *S. cerevisiae*, our predictions are based indirectly on ecological findings for *S. paradoxus* and laboratory growth experiments from North American strains (Sweeney et al. 2004). In using this laboratory estimate, we assume that the physiological response to temperature is fixed within species. However, the *S. paradoxus* strains used by Sweeney et al. (2004) have a North American genotype (Kuehne et al. 2007) that suggests they could have higher optimum growth temperature than *S. paradoxus* with European genotypes (Leducq et al. 2014, 2015). We may therefore underestimate the difference between *S. cerevisiae* and *S. paradoxus* (Leducq et al. 2014). Another laboratory estimate however, suggests that we could be using an overestimate (Salvadó et al. 2011; see Data S1 for discussion). Thus, the optimum summer temperature range that we predict for *S. cerevisiae* needs to be tested by directly sampling trees in subtropical and tropical regions with precise site locations and trunk girth measurements.

Another important predictor we uncover here for *S. paradoxus* isolation frequency is tree trunk girth (Fig. 2), which is consistent with the intuitive notion that older trees harbor a greater diversity of microbial species including yeast. Indeed, the effect of trunk girth is so strong that if we had not included trunk girth in our model, we would not have detected an association of *S. paradoxus* isolation frequency with temperature. Intriguingly, the possible accumulation of yeasts on oak trees as they grow suggests a process of microbial succession that could parallel below ground processes (Bardgett 2005; Bardgett et al. 2005). Only 42% of the deviance we observed in *S. paradoxus* isolation frequency could be explained by trunk girth and $T_{\max}$ together, suggesting that there are other important predictors of *S. paradoxus* isolation frequency that we do not study here. For example, *S. paradoxus* abundance could be influenced by interactions with other microbes (Kowallik et al. 2015); the availability of nutrients (Sampaio and Gonçalvez 2008), water or oxygen (Deak 2006); acidity (Deak 2006) or sampling season (Glushakova et al. 2007; Charron et al. 2014).

The general caveats that apply when considering climate envelope models (Araújo and Peterson 2012; Jarnevich et al. 2015) also apply to our findings. We outline regions that have summer temperatures predicted to be associated with high *S. paradoxus* or *S. cerevisiae* isolation frequency (Fig. 4). We do not suggest that these regions show the actual distribution of the species however, because they might not contain viable habitat (Araújo and Peterson 2012; Jarnevich et al. 2015).

Our results also show that *S. paradoxus* and *S. cerevisiae* are not the only oak‐associated yeast species with geographic distributions in Europe that could be associated with temperature (Table 1). *W. anomalus* is relevant to humans, as a wine yeast, food spoilage yeast and biocontrol agent (Passoth et al. 2006), occurring naturally on plants, and soil (Kurtzman 2011). This species can be found on trees in northern North America (Charron et al. 2014; Sylvester et al. 2015) and on central European mountains (Sláviková et al. 2007). We present evidence that *W. anomalus* is more common on northern than on southern European oaks (Table 1), suggesting a southern limit to its distribution in European woodlands. Such a conclusion is consistent with the finding that *W. anomalus* is more often isolated by incubating bark at low than at high temperatures (10°C vs. 30°C; Sylvester et al. 2015). *L. thermotolerans* also naturally occurs on oak bark (Sampaio and Gonçalvez 2008; Charron et al. 2014; Freel et al. 2015; Sylvester et al. 2015) and fruit (Lachance and Kurtzman 2011), and has been proposed as a good model species for yeast population genetics (Freel et al. 2014, 2015). We find that it is more abundant on oaks in southern Europe (Table 1), consistent with the finding that it is isolated from bark at high temperatures (30°C vs. 10°C; Sylvester et al. 2015).

Knowledge of the climate associations of animal and plant species can lead to the discovery of new populations, as well as the prediction of glacial refugia, biodiversity hotspots, extinction risks, and responses to climate change (Araújo and Peterson 2012; Jarnevich et al. 2015). Because they are too small to see, geographic distributions and therefore ecological associations are more difficult to determine for free‐living microbes. However for microbial species that can be cultured, ecologically relevant factors such as temperature preferences are easier to determine experimentally than they are for plants or animals. Our work suggests that laboratory estimates of optimum growth temperature could be used to predict global distributions of free‐living microbes.

## Conflict of Interest

None declared.

## Data Accessibility

DNA sequences determined for this study are available in GenBank: KT206983‐KT207282. Photographs of host plants and DNA sequences that did not fulfill the submission criteria at GenBank are available together with all data at https://github.com/bensassonlab/yeastecology/.

## Supporting information


**Figure S1.** Approximate geographic positions of 81 *S. cerevisiae* strains from China are close to locations with expected summer temperatures.

**Data S1.** Supplemental Results
**Table S1.** Primers used to identify yeast species by PCR and DNA sequencing.

**Data S2.** Summarises the BLAST results for the 371 DNA sequences generated for this study, the species call of the associated yeast strains, and NCBI accession numbers.

**Data S3.** Summarises the presence or absence of *S. cerevisiae* (Scer), *S. paradoxus* (Spar), other yeast that is amplified by primers in the ITS region (otherAmplifiedITS), or other microbial growth (otherGrowth) for every sample collected for this study.

**Data S4.** Details of 301 *S. cerevisiae* and 246 *S. paradoxus* isolates and the geographic locations from which they were sampled.
