# Supplementary figures and images for: Summer temperature can predict the distribution of wild yeast populations
Source: Ecol Evol. 2016 Jan 27;6(4):1236–50. doi: 10.1002/ece3.1919 (PMC4761769; doi:10.1002/ece3.1919)

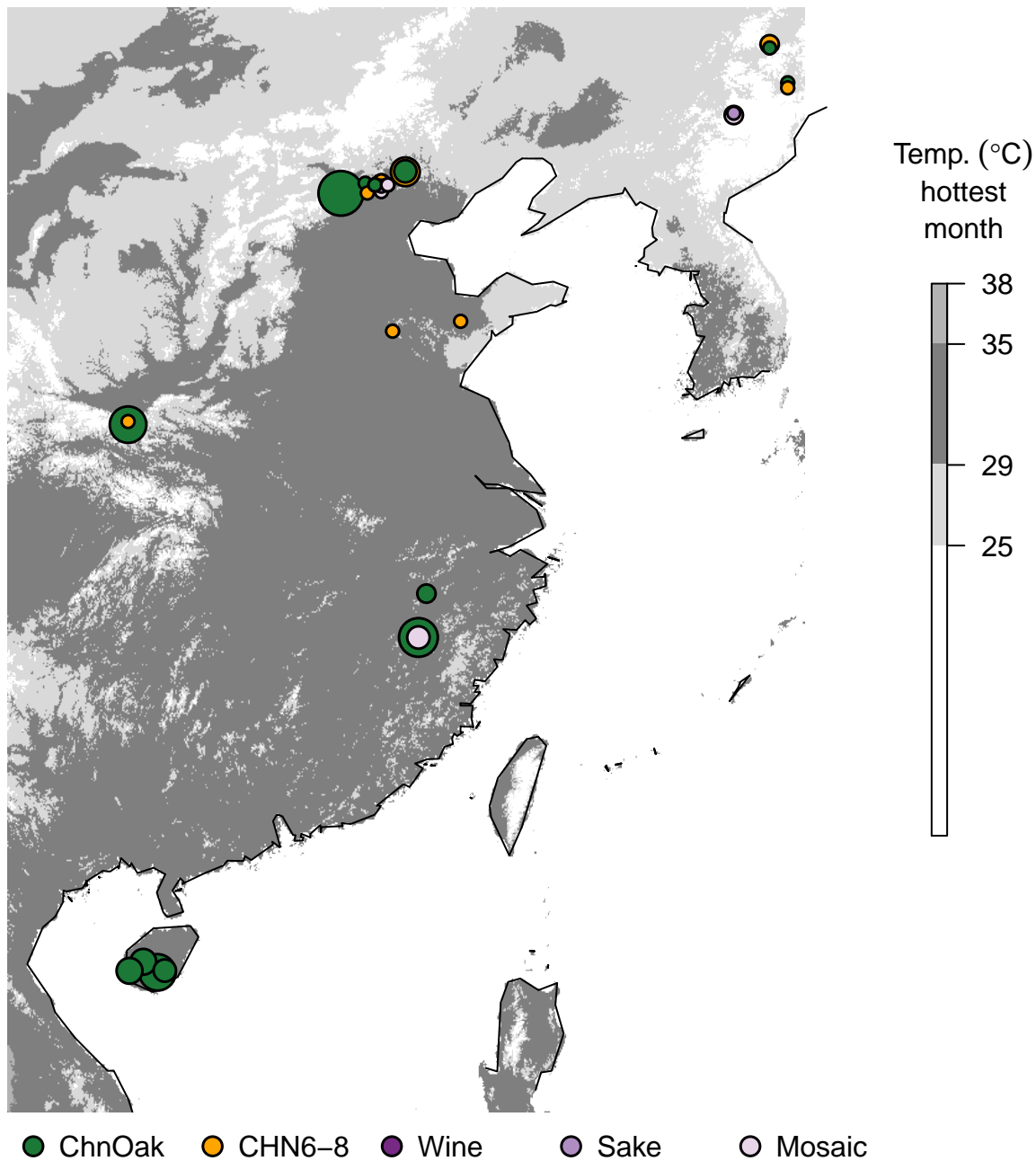

Supplement: Supplementary file 1 — Figure S1. Approximate geographic positions of 81 S. cerevisiae strains from China are close to locations with expected summer temperatures. [file ECE3-6-1236-s001.pdf]
