# Supplementary material for: Summer temperature can predict the distribution of wild yeast populations
Source: Ecol Evol. 2016 Jan 27;6(4):1236–50. doi: 10.1002/ece3.1919 (PMC4761769; doi:10.1002/ece3.1919)
Supplement: Supplementary file 2 — Data S1. Supplemental Results Table S1. Primers used to identify yeast species by PCR and DNA sequencing. [file ECE3-6-1236-s002.pdf]

Supplemental Results

Identification of *Candida albicans* from oak trees in the New Forest, UK

The human commensal and pathogenic yeast species *Candida albicans* has only rarely been isolated from natural environments that are not associated with mammals (Tanghe *et al.*, 2005; Lachance *et al.*, 2011; Maganti *et al.*, 2011). Here we isolated three yeast strains from northern European oak trees with rDNA sequences, identical to several different isolates of *C. albicans* (site 6 in Figure 1 and Table 2) that were isolated from humans around the world (Supplemental File 2). All three rDNA sequences from these strains differed (by at least 15 nucleotides) from the type strain of the most closely related species *C. dubliniensis* (Lachance *et al.*, 2011) suggesting that these are indeed *C. albicans*, and that it occurs in this natural habitat. We note that *C. albicans* was isolated from three of the oldest oak trees sampled in this study (trunk girth, 2.8-4.1m).

A few lines of evidence suggest that these strains do not represent human commensal contaminants introduced during sample processing. To avoid sample contamination (and exposure of workers to unidentified microbes), all samples were processed in a class II cabinet. Additionally, negative controls generated in the field generated no colonies after enrichment culturing. Similarly, all negative controls associated with DNA extraction and PCR amplification of these samples were also blank, and identical rDNA sequences were generated by two different lab members. Two of the rDNA sequences associated with the three different *C. albicans* differ by an unambiguous single nucleotide insertion or deletion, therefore at least 2 distinct contaminants would be needed to explain this diversity.

If ancient oaks do indeed represent a natural habitat of *C. albicans* however, it must be rare in this environment because we isolated it from only one site out of 13. Furthermore,

others have isolated other *Candida* species from trees but not *C. albicans*, suggesting that they could have recovered *C. albicans* if it was present at appreciable frequencies (Maganti *et al.*, 2011; Charron *et al.*, 2014; Sylvester *et al.*, 2015)

The ecological niche of *S. cerevisiae*

In order to test whether *S. cerevisiae* is more abundant on fruits than on other substrates, we compared *S. cerevisiae* isolation rates between fermenting grape must (n = 12), grapes (n = 24), grape vine bark (n = 21) and oak bark (n = 4) samples in two UK vineyards after the grape harvest. As expected, *S. cerevisiae* is isolated more readily from fermenting grape must samples (67%) than it is from grape samples (21%; Fisher's exact test, P = 0.01), but *S. cerevisiae* isolation rates from grapes are very similar to those for grapevine bark (29%; Fisher's exact test, P = 0.7) and oak bark (25%; Fisher's exact test, P = 1).

Likewise, fig trees in Southern Europe showed similar *S. cerevisiae* isolation rates from fig tree bark (1 out of 9, 11%) and figs (5 out of 84, 6%; Fisher's exact test, P = 0.5). Although it was difficult to quantify, we noticed that unripe figs may have lower rates of yeast isolation, and our sampling of unripe fruit could contribute to the low overall *S. cerevisiae* rate from figs. Fig tree bark may therefore provide a more stable substrate for yeast isolation, especially where trees are old.

Differences among trees in the prevalence of *Saccharomyces paradoxus*

This study generated data for 604 bark samples from 126 different oak trees that were tested for the presence or absence of *S. paradoxus* by enrichment culture at 25 or 30°C. In most cases (87 trees), 4 pieces of bark were sampled from the same tree, and 28 trees were re-visited in different months (June-September, and November) and years (2006-2011).

To test whether the probability of isolating *S. paradoxus* differs among trees, and for possible effects of sample weight or time of collection, we used logistic regression (GLM with binomial errors) to model the presence or absence of *S. paradoxus* in each bark sample (a binary response variable). The initial model included a factor to describe the effect of each oak tree (126 levels), a second factor for collection month (5 levels: June-September, November), weight (in grams) as a continuous variable, and the two-way interaction between weight and collection month. This full initial model was simplified by subtracting terms in a stepwise manner starting from the two-way interaction and testing whether each subtraction resulted in a worse model using χ^2 tests as recommended in Crawley (2005).

In this analysis, bark weight was not a good predictor of the presence or absence of *S. paradoxus* (GLM, -0.01% deviance, $d.f. = 1, P = 0.8$). There was also no obvious difference in isolation rates in different months (GLM, -1% deviance, $d.f. = 4, P = 0.1$). This can be explained by the fact that most samples (75%) were collected between 25th August and 7th September. There were, however, clear differences among trees in the likelihood of *S. paradoxus* isolation from their bark, which explain 52% of the variation among samples (GLM, $\chi^2 =, d.f. = 125, P = 2 \times 10^{-9}$).

Because weight and collection month do not have a significant effect on *S. paradoxus* isolation frequency, we were able to pool data for multiple bark samples from each oak tree in the main analysis, and therefore to use the proportion of bark samples from each tree that resulted in *S. paradoxus* isolates as a response variable (see Methods). This was preferable to using a binary response variable with several different explanatory variables, which would result in fitted probabilities of zero or one in the final model, and these lead to known problems with binomial GLMs (Venables and Ripley, 2002).

Choice of laboratory study to estimate the difference between *S. paradoxus* and *S. cerevisiae* in optimal growth temperature

We rely on an accurate estimate in the difference between optimal growth, but studies that compare *S. paradoxus* and *S. cerevisiae* measure the difference approximately (Liti *et al.*, 2009; Leducq *et al.*, 2014) or disagree in the extent of the difference (2°C, Salvadó *et al.*, 2011; 7°C, Sweeney *et al.*, 2004). We chose to use the study by Sweeney *et al.* (2004) because (i) they screen many strains from both *S. paradoxus* and *S. cerevisiae* that were isolated from oak trees and oak trees are also the focus of this study; (ii) their growth profiles are typical for each species (Liti *et al.*, 2009); and (iii) their estimate is closer to the observation by Leducq *et al.* (2014) that type strains for *S. cerevisiae* and *S. paradoxus* differ by approximately 10°C. In addition, the *S. paradoxus* strains used by Sweeney *et al.* (2004) have a North American genotype (*SpB*; Kuehne *et al.*, 2007) that suggests they may grow better at high temperatures than *S. paradoxus* strains with a European genotype (Leducq *et al.*, 2014, 2015). It is therefore possible that Sweeney *et al.* (2004) underestimate the difference between *S. cerevisiae* and *S. paradoxus* in optimum growth temperature.

How would our results differ if we had chosen to use a different estimate (Salvadó *et al.*, 2011) from fewer strains of *S. paradoxus* and *S. cerevisiae* mostly isolated from fruit? Salvadó *et al.* (2011) report a *S. cerevisiae* optimum 2°C higher than *S. paradoxus* and a maximum temperature for growth that is 4°C higher. If we assume a 4°C difference, then this correctly predicts the presence of *S. cerevisiae* isolates with wild genotypes, but does not explain why only human-associated strains or those approximately mapping to mountains are isolated between 22°C and 25°C (Figure 5, Supplemental Figure 1) or why no *S. cerevisiae* were isolated in the southern UK (23°C, Johnson *et al.*, 2004) or Canada (T_{max} 25°C, Charron *et al.*, 2014).

Supplemental Tables and Figures

Supplemental Table 1: **Primers used to identify yeast species by PCR and DNA sequencing**

Species	Locus	PCR name	Primer name	Primer sequence
Saccharomycetales	rDNA[a]	ITS	ITSf1	AAAGTCGTAACAAGGTTTCCGTAG
			ITSr	TCCGCTTATTGATATGCTTAAGTTC
		ITS2	ITSf2	GAAsTAAAAGTCGTAACAAGGTTTCC
			ITSr	TCCGCTTATTGATATGCTTAAGTTC
		S1L	S1Lf	ATTTGAGGTCAAACTTTAAGAACATTG
			S1Lr	AGAGGAACTAAAAGTCGTAACAAGG
Saccharomyces	CEN6	cepC6[b]	cepC6f	GAACCTGTCGAAGTTGTTAATGC
			cepC6r	TrGAAGGTTTCTTTGGwGCCAT
		parC6[c]	parC6f	CGAAGTTGTTAATGCGAAATATTCTA
			parC6r	ACCTCTCTTCTCAAAGTTTGCCT
S. paradoxus	CEN9	parC9	parC9f	ATATTCTAGCCGATCTGGAACTTG
			parC9r	CAGATAACGTTAAAAGTTCTGTCCAA
	CEN15	parC15	parC15f	TCATATTTATAACAAGCGATCAAAGC
			parC15r	GAAGTACATCCTGATTTTTAGAAGCC

See Bensasson (2011) for details of the three primer pairs (cerC6, cerC9 and cerC15) used for *S. cerevisiae*-specific amplification of centromere loci.

[a] Primers anneal to 18SrRNA and 25SrRNA, and amplify all of ITS1-5.8SrRNA-ITS2 (see Methods).

[b] Amplifies *S. cerevisiae*, *S. paradoxus*, and *S. kudriavzevii* DNA, but with a product of a different size for *S. kudriavzevii*.

[c] Amplifies *S. paradoxus* and *S. kudriavzevii* DNA, but with a product of a different size for *S. kudriavzevii*.

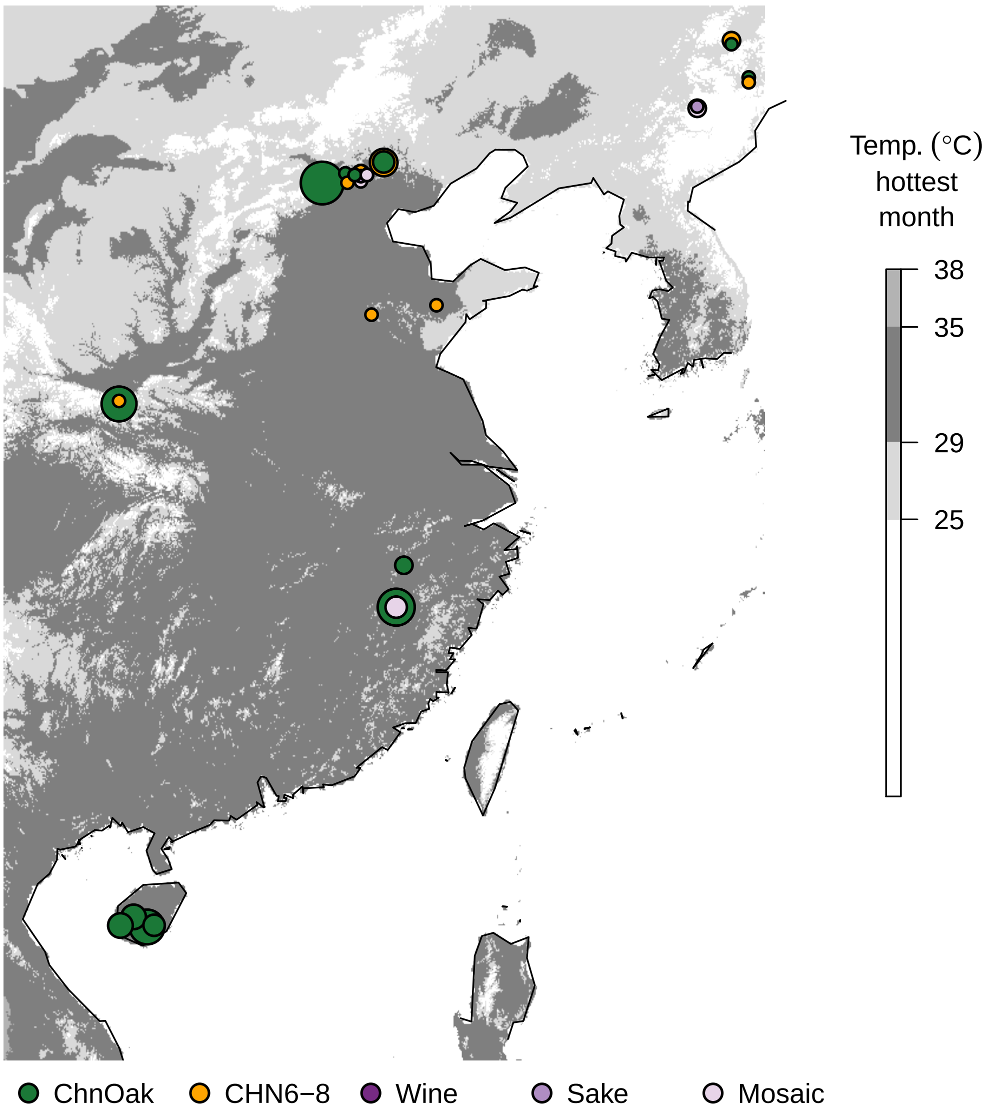

Supplemental Figure 1: **Approximate geographic positions of 81 *S. cerevisiae* strains from China are close to locations with expected summer temperatures.** Regions shaded in grey show summer temperatures that we predict are optimal for *S. cerevisiae*. Approximate collection sites described in Wang *et al.* (2012) are shown with points scaled by the square root of sample size. Strains with genotypes that are associated with humans are shown in purple, and mosaic strains show recent admixture between multiple populations. Strains with genotypes that are so far only associated with woodlands are shown in green (CHNI, CHNII, CHNIII, CHNIV, CHNV), strains with genotypes that are more similar to those seen in other parts of the world are shown in orange (CHNVI, CHNVII, CHNVIII) and all of the approximate isolation sites for these are close to locations with the optimum summer temperature (T_{max}) for *S. cerevisiae*.

References

Bensasson, D. (2011). "Evidence for a high mutation rate at rapidly evolving yeast centromeres." *BMC Evol Biol*, **11**: 211.

Charron, G., Leducq, J.B., Bertin, C., Dubé, A.K., and Landry, C.R. (2014). "Exploring the northern limit of the distribution of *Saccharomyces cerevisiae* and *Saccharomyces paradoxus* in North America." *FEMS yeast research*, **14**(2): 281–288.

Crawley, M.J. (2005). *Statistics: An Introduction Using R*. Wiley-Blackwell, Chichester, West Sussex, England, 1 edition edition. ISBN 978-0-470-02298-6.

Johnson, L.J., Koufopanou, V., Goddard, M.R., Hetherington, R., Schafer, S.M., and Burt, A. (2004). "Population Genetics of the Wild Yeast *Saccharomyces paradoxus*." *Genetics*, **166**(1): 43–52.

Kuehne, H.A., Murphy, H.A., Francis, C.A., and Sniegowski, P.D. (2007). "Allopatric divergence, secondary contact, and genetic isolation in wild yeast populations." *Curr Biol*, **17**(5): 407–11.

Lachance, M.A., Boekhout, T., Scorzetti, G., Fell, J.W., and Kurtzman, C.P. (2011). "Chapter 90 - *Candida* Berkhout (1923)." In C.P.K.W.F. Boekhout, editor, "The Yeasts (Fifth Edition)," pages 987–1278. Elsevier, London. ISBN 978-0-444-52149-1.

Leducq, J.B., Charron, G., Samani, P., Dubé, A.K., Sylvester, K., James, B., Almeida, P., Sampaio, J.P., Hittinger, C.T., Bell, G., and Landry, C.R. (2014). "Local climatic adaptation in a widespread microorganism." *Proceedings of the Royal Society B: Biological Sciences*, **281**(1777): 20132472.

Leducq, J.B., Nielly-Thibault, L., Charron, G., Eberlein, C., Verta, J.P., Samani, P., Sylvester, K., Hittinger, C.T., Bell, G., and Landry, C.R. (2015). "Speciation driven by hybridization and chromosomal plasticity in a wild yeast." *bioRxiv*, page 027383.

Liti, G., Carter, D.M., Moses, A.M., Warringer, J., Parts, L., James, S.A., Davey, R.P., Roberts, I.N., Burt, A., Koufopanou, V., Tsai, I.J., Bergman, C.M., Bensasson, D., O/'Kelly, M.J.T., van Oudenaarden, A., Barton, D.B.H., Bailes, E., Nguyen, A.N., Jones, M., Quail, M.A., *et al.* (2009). "Population genomics of domestic and wild yeasts." *Nature*, **458**(7236): 337–341.

Maganti, H., Bartfai, D., and Xu, J. (2011). "Ecological structuring of yeasts associated with trees around Hamilton, Ontario, Canada." *FEMS yeast research*, **12**: 9–19.

Salvadó, Z., Arroyo-López, F.N., Guillamón, J.M., Salazar, G., Querol, A., and Barrio, E. (2011). "Temperature Adaptation Markedly Determines Evolution within the Genus *Saccharomyces*." *Applied and Environmental Microbiology*, **77**(7): 2292–2302.

Sweeney, J.Y., Kuehne, H.A., and Sniegowski, P.D. (2004). "Sympatric natural *Saccharomyces cerevisiae* and *S. paradoxus* populations have different thermal growth profiles." *FEMS Yeast Research*, **4**(4-5): 521–525.

Sylvester, K., Wang, Q.M., James, B., Mendez, R., Hulfachor, A.B., and Hittinger, C.T. (2015). "Temperature and host preferences drive the diversification of Saccharomyces and other yeasts: a survey and the discovery of eight new yeast species." *FEMS yeast research*, **15**(3): fov002.

Tanghe, A., Carbrey, J.M., Agre, P., Thevelein, J.M., and Van Dijck, P. (2005). "Aquaporin expression and freeze tolerance in *Candida albicans*." *Applied and Environmental Microbiology*, **71**(10): 6434–6437.

Venables, W. and Ripley, B. (2002). *Modern Applied Statistics with S*, volume 4th. Springer, New York.

Wang, Q.M., Liu, W.Q., Liti, G., Wang, S.A., and Bai, F.Y. (2012). "Surprisingly diverged populations of *Saccharomyces cerevisiae* in natural environments remote from human activity." *Molecular Ecology*, **21**(22): 5404–5417.
